# Through-and-Through Dissection of the Soft Palate for Pharyngeal Flap Inset: A “Good-Fast-Cheap” Technique for Any Etiology of Velopharyngeal Incompetence

**DOI:** 10.1177/10556656211021738

**Published:** 2021-06-17

**Authors:** Michael Carr, Michaela Skarlicki, Sheryl Palm, Marija Bucevska, Jeffrey Bone, Arun K. Gosain, Jugpal S. Arneja

**Affiliations:** 1 Division of Plastic Surgery, Department of Surgery, University of British Columbia, Vancouver, BC, Canada; 2 School of Nutrition, Ryerson University, Toronto, Canada; 3 Department of Audiology & Speech, University of British Columbia, Cleft Palate-Craniofacial Clinic, British Columbia Children’s Hospital, Vancouver, BC, Canada; 4 Department of Obstetrics and Gynecology, University of British Columbia, Vancouver, BC, Canada; 5 Division of Pediatric Plastic Surgery, Lurie Children’s Hospital, Northwestern University Feinberg School of Medicine, Chicago, USA; 6 Sauder School of Business, University of British Columbia, Vancouver, BC, Canada

**Keywords:** surgical technique, pharyngeal flap, velopharyngeal incompetence

## Abstract

**Objective:**

To determine the efficacy and resource utilization of through-and-through dissection of the soft palate for pharyngeal flap inset for velopharyngeal incompetence (VPI) of any indication.

**Design:**

Retrospective review.

**Setting:**

Tertiary care center.

**Patients:**

Thirty patients were included. Inclusion criteria were diagnosis of severe VPI based on perceptual speech assessment, confirmed by nasoendoscopy or videofluoroscopy; VPI managed surgically with modified pharyngeal flap with through-and-through dissection of the soft palate; and minimum 6 months follow-up. Patients with 22q11.2 deletion syndrome were excluded.

**Intervention:**

Modified pharyngeal flap with through-and-through dissection of the soft palate.

**Main Outcome Measure(s):**

Velopharyngeal competence and speech assessed using the Speech-Language Pathologist 3 scale.

**Results:**

The median preoperative speech score was 11 of 13 (range, 7 to 13), which improved significantly to a median postoperative score of 1 of 13 (range 0-7; *P* < .001). Velopharyngeal competence was restored in 25 (83%) patients, borderline competence in 3 (10%), and VPI persisted in 2 (7%) patients. Complications included 1 palatal fistula that required elective revision and 1 mild obstructive sleep apnea that did not require flap takedown. Median skin-to-skin operative time was 73.5 minutes, and median length of stay (LOS) was 50.3 hours.

**Conclusions:**

This technique allows direct visualization of flap placement and largely restores velopharyngeal competence irrespective of VPI etiology, with low complication rates. Short operative time and LOS extend the value proposition, making this technique not only efficacious but also a resource-efficient option for surgical management of severe VPI.

## Introduction

Velopharyngeal incompetence (VPI) is a condition in which the velopharyngeal sphincter (VPS) fails to create a functional seal between the oral and nasal cavities during phonation. Incomplete closure during speech leads to air escape causing hypernasality, increased nasal resonance, and decreased intelligibility (Ruda et al., 2012). Furthermore, children with untreated VPI may develop secondary sequelae, including compensatory speech misarticulations (glottal stops and pharyngeal fricatives), softness of voice, and laryngeal nodules due to increased laryngeal airflow (Hogan, 1973; Kummer et al., 2015; de Blacam et al., 2018). Decreased intelligibility from VPI adversely affects patients’ schooling, work, and psychosocial health and negatively impacts children’s quality of life (Sloan, 2000; Leclerc et al., 2014). Surgery is the only effective intervention for severe VPI related to anatomical deficiencies and works by creating a functional seal between the oropharynx and nasopharynx during phonation.

The pharyngeal flap is the most described surgical technique for the management of VPI, designed to create an incomplete central velopharyngeal obstruction that permits peripheral airflow between the oral and nasal cavities during phonation (Setabutr et al., 2015; de Blacam et al., 2018). Since Schönborn (1875) first described an inferiorly based pharyngeal flap, the pharyngeal flap has been a mainstay in speech surgery. The technique has been revised many times, by the likes of Rosenthal (1924), Padgett (1930), Sanvenero-Roselli (1935), and Conway (1951), finally leading to the description of the lateral port control flap by Hogan (1973)—a technique that is considered by many as a first-line technique for VPI management for its consistent speech outcomes and relatively low complication rates (Boutros & Cutting, 2013).

Though commonly used, the pharyngeal flap is not without its criticisms, even with abundant refinement over the years: respiratory obstruction, flap dehiscence, and insufficient velopharyngeal obturation caused by raising a flap of inadequate height, are commonly cited challenges related to the pharyngeal flap (Shprintzen et al., 1979; Valnicek et al., 1994; Witt et al., 1998; Hofer et al., 2002). For these reasons, surgeons have continued to report new pharyngeal flap designs, evolving from the first suggestion of “tailor-made” flaps in 1979 (Shprintzen et al., 1979).

Arneja et al. (2008) previously described a pharyngeal flap technique for the treatment of VPI in children with 22q11.2 deletion syndrome (22qDS), employing through-and-through dissection of the soft palate to achieve high flap inset. This flap design was intended to address previously reported criticisms of the pharyngeal flap, namely, high rates of flap dehiscence, insufficient VPS obturation due to the challenges of raising flaps of sufficient height, and the technical difficulties of limited visualization of the posterior soft palate during flap inset (Shprintzen et al., 1979; Valnicek et al., 1994; Huang et al., 1998; Witt et al., 1998; Hofer et al., 2002; Emara & Quriba, 2012). They showed that in a series of 8 patients with VPI related to 22qDS, this technique effectively restored velopharyngeal competence in all patients, with no cases of flap dehiscence, respiratory complication, or revision surgery.

In its simplest sense, surgery is a collection of resources (human, technology, equipment, physical plant, materials, etc) utilized to produce a desired surgical outcome. In industrial production, the ideal outcome is often a trade-off between good (quality), fast (time), and cheap (cost), with firms having a difficult time achieving all 3 (Atkinson, 1999). In an era of increased focus on fiscal prudence and cost control in health care, it is critical for surgeons to look at these metrics when evaluating the outcomes of our interventions.

Herein, the present report describes the same modified pharyngeal flap technique used to treat patients with VPI from etiologies other than 22qDS. We hypothesize that the improved control for pharyngeal flap inset will confer good functional outcomes with low complication rates for treatment of VPI in all etiologies, not only those related to 22qDS. Functional, surgical, clinical, and resource utilization outcomes in patients with severe VPI not related to 22qDS over the past 6 years are reviewed.

## Patients and Methods

### Data Collection

A retrospective review was conducted of patients with severe VPI who underwent modified pharyngeal flap with through-and-through dissection of the soft palate, as described previously (Arneja et al., 2008). Data evaluated were patient age at the time of surgery, gender, palate anatomy, etiology of VPI, clinical and surgical assessments, multiview videofluoroscopy and nasoendoscopy data, perceptual speech scores, operative times, and complication rates.

Inclusion criteria were a diagnosis of severe VPI based on perceptual speech assessment, with confirmation by nasoendoscopy or videofluoroscopy; surgical management of VPI by modified pharyngeal flap with through-and-through dissection of the soft palate; and a minimum follow-up of 6 months with a complete postoperative speech assessment. We excluded all patients with 22qDS as we have previously published on those outcomes.

Speech was assessed by a trained speech and language pathologist using Speech-Language Pathologist 3 (SLP-3), a standardized speech/voice rating scale. This scale perceptually evaluates articulation, facial grimace, nasal air emission (hypernasality), laryngeal resonance (hoarseness/breathiness), and nasal resonance, with a total possible score of 13. A score of 7 or greater indicates an incompetent valving mechanism, a score of 3 or less suggests normal velopharyngeal competence, and scores between 3 and 7 are considered borderline competence (Denny et al., 1993).

Multiview videofluoroscopy and nasoendoscopy of the velopharyngeal port during speech production qualitatively assessed palatal movement, lateral wall movement, and the size of gap between the velum and the posterior pharyngeal wall. Lateral wall movement was reported as either none, slight, or moderate, and velopharyngeal gap size as small, medium, or large, corresponding to 0.8 to 0.9, 0.5 to 0.7, and 0.0 to 0.4 gap size ratios, respectively. These measurements were used to determine a diagnosis of VPI by our cleft team’s SLP.

The operative records were reviewed to calculate skin-to-skin surgical time and anesthesia time (“wheels in to wheels out time”), and these data were used to calculate a case cost, using previously published bottom-up microcosting analysis (Malic et al., 2014). We excluded the time required for tympanostomy tubes, as not all patients in our series required this at the time of pharyngeal flap.

### Surgical Technique

The surgical technique described in this present series is largely the same as reported previously by Arneja et al. (2008) with 3 minor modifications: (1) rather than tunnel the flap through a fishmouth incision and counterincision in the soft palate, we use a direct transverse incision through the soft palate, approximately 3 to 4 mm posterior to the hard–soft junction; (2) the posterior pharyngeal wall donor site is closed primarily, except for the most superior aspect proximal to the flap as to avoid compression of the pedicle base; and (3) nasal stents are not used, as the posterior pharyngeal wall is closed primarily, thereby reducing the risk of forming postoperative synechiae between the demucosalized surfaces of the lateral pharyngeal ports.

### Outcome and Statistical Analysis

Pre- and postoperative speech scores were presented with side-by-side boxplots. To determine statistical significance in pre- and postoperative scores, paired *t* tests were used and 95% CIs and *P* values for the difference are reported. These analyses were checked for robustness from normality assumptions by the Wilcoxon rank test (not included here). The 4 components of the speech score were analyzed similarly, with the *P* value being adjusted by the standard Bonferroni correction (division by 4 in this case). To determine the effects of various patient demographics on the score differences, data were plotted via side-by-side boxplots and scatterplots. Furthermore, linear models were used for the postoperative score, with adjustment for each possible demographic or clinical predictor, and the preoperative score. Each of these predictors was included independently, as there were not sufficient degrees of freedom to use multivariate models. In all models, .05 was used for statistical significance and 95% CIs for the regression coefficients were reported. We divided complications to minor (snoring, dehiscence, infection, feeding difficulties) and major (obstructive sleep apnea [OSA], fistula requiring reoperation, hemorrhage, airway obstruction, reintubation, readmission, mortality). This study was approved by the University of British Columbia Children's & Women's Research Ethics Board, approval# H18-01137. Patient consent was not required.

## Results

Thirty patients met our inclusion criteria with a median age of 5.5 years at the time of surgery (range 3.8-16.7 years), 17 were male and 13 were female. The VPI was mostly related to postcleft palate repair (20/30); other etiologies included submucous cleft palate (3/30), neurologic VPI (3/30), and idiopathic noncleft VPI (4/30). Eleven patients had concomitant syndromic diagnoses, which included Pierre Robin syndrome (3), Opitz syndrome (1), developmental coordination disorder (2), Stickler syndrome, mitochondrial encephalopathy, lactic acidosis, and stroke-like episodes (MELAS) (1), KBG syndrome (1), or complex neurodevelopmental disorders (3). All patients had severe VPI with the majority having moderate or large pharyngeal gap size (25/30), and none or slight lateral pharyngeal wall mobility (26/30) by nasoendoscopy or videofluoroscopy. Demographic data and preoperative palate dynamics are summarized in Table 1.

**Table 1.** Summary of Patient Demographics, Preoperative Velopharyngeal Dynamics, and Speech Therapy.

Characteristic	Value	Mean difference in postoperative SLP score (95% CI)^a^	*P* value
Sex			
Male	17 (0.57)		
Female	13 (0.43)		
Age (years)			
Mean (SD)	6.7 (3.35)	−0.11 (−0.35 to 0.14)^b^	.38
Range	3.8-16.7		
Etiology of VPI			
Cleft lip or palate	20 (0.67)	Reference	Reference
Submucous cleft palate	3 (0.10)	−0.62 (−3.53 to 2.3)	.67
Neurologic VPI	3 (0.10)	−0.76 (−3.39 to 1.86)	.55
Other noncleft VPI	4 (0.13)	−1.07 (−3.39 to 1.24)	.35
Syndromic diagnosis			
Present	11 (0.37)	−0.1 (−1.66 to 1.46)	.9
Absent	19 (0.63)	Reference	
Previous oropharyngeal surgery			
Yes	21 (0.70)	0.8 (−0.93 to 2.54)	.35
No	9 (0.30)	Reference	Reference
Velopharyngeal closure pattern^c^			
Coronal	9 (0.30)	Reference	Reference
Circular	19 (0.63)	−0.36 (−2.03 to 1.31)	.66
Circular with Passavant ridge	2 (0.07)	NA	
Sagittal	0	NA	
Velopharyngeal gap size			
Small	5 (0.17)	Reference	Reference
Moderate	11 (0.37)	−0.8 (−3.25 to 1.65)	.51
Large	14 (0.47)	−1.34 (−3.85 to 1.17)	.28
Lateral wall motion			
None	9 (0.30)	Reference	Reference
Slight	17 (0.57)	−0.47 (−2.16 to 1.23)	.58
Moderate	4 (0.13)	0.81 (−1.65 to 3.27)	.51
Postoperative speech therapy			
Yes	27 (0.90)	−5.53 (−6.8 to −4.27)	<.001
No	3 (0.10)	Reference	Reference

Abbreviations: NA, not applicable; SLP, Speech-Language Pathologist; VPI, velopharyngeal incompetence.

^a^ Adjusted for preoperative score.

^b^ Per 1 year change.

^c^ Comparison is for coronal versus other.

**Figure 1.**
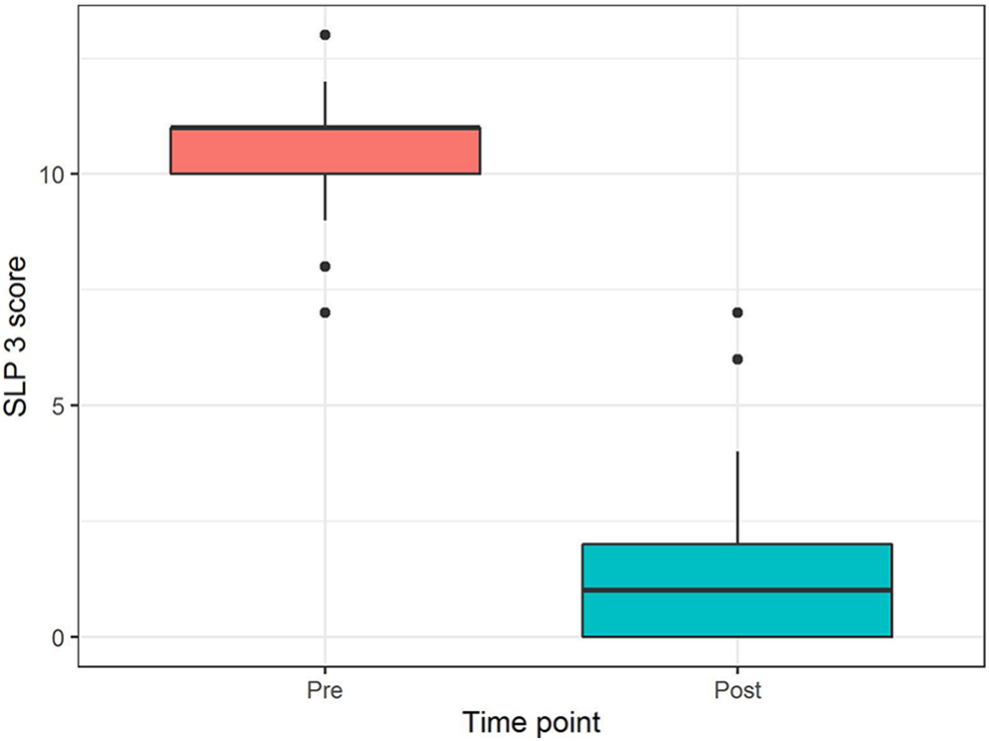
Comparison of pre- and postoperative perceptual speech scores, presented with side-by-side boxplots. Mean postoperative scores improved significantly (*P* < .001).

Pre- and postoperative speech scores for all 30 patients are summarized in Figure 1. The median preoperative speech score was 11 (range 7-13), which improved significantly (*P* < .001) to a median postoperative score of 1 (range 0-7), at a median follow-up time of 16 months (range 6-50 months). Two patients had persistent VPI following their initial surgery (SLP-3 score >6). Two of these patients had not received adequate speech therapy. The third patient had a palatal fistula that underwent revision surgery, subsequent to which VPI competence was completely restored.

**Figure 2.**
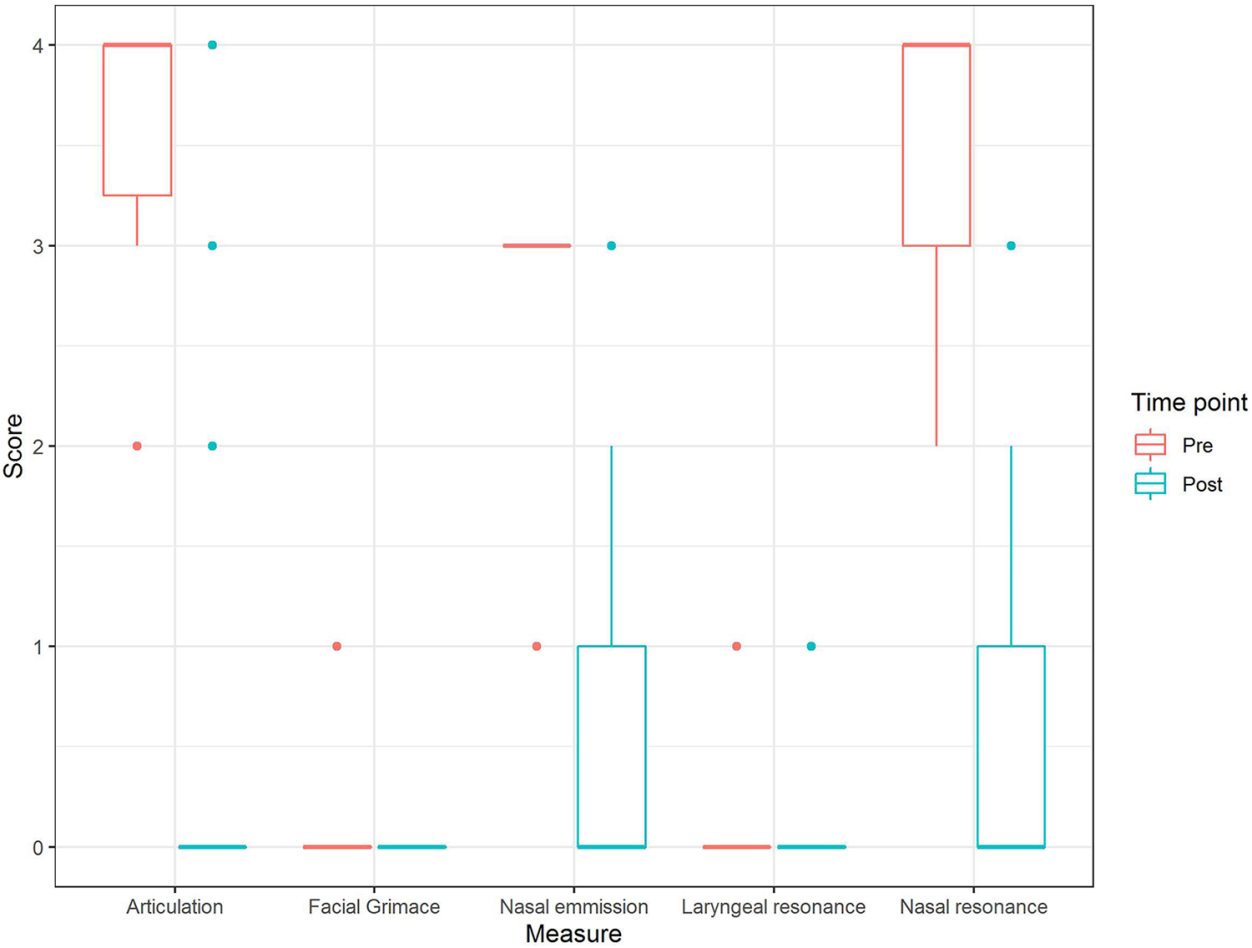
Changes in individual components of SLP-3 perceptual speech score after surgery.

The effects of treatment on independent components of the SLP-3 score (articulation, facial grimace, nasal air emission, laryngeal resonance, and nasal resonance) were assessed and are presented in Figure 2. Except for laryngeal resonance, each speech component showed significant improvement following surgery (*P* < .01).

**Figure 3.**
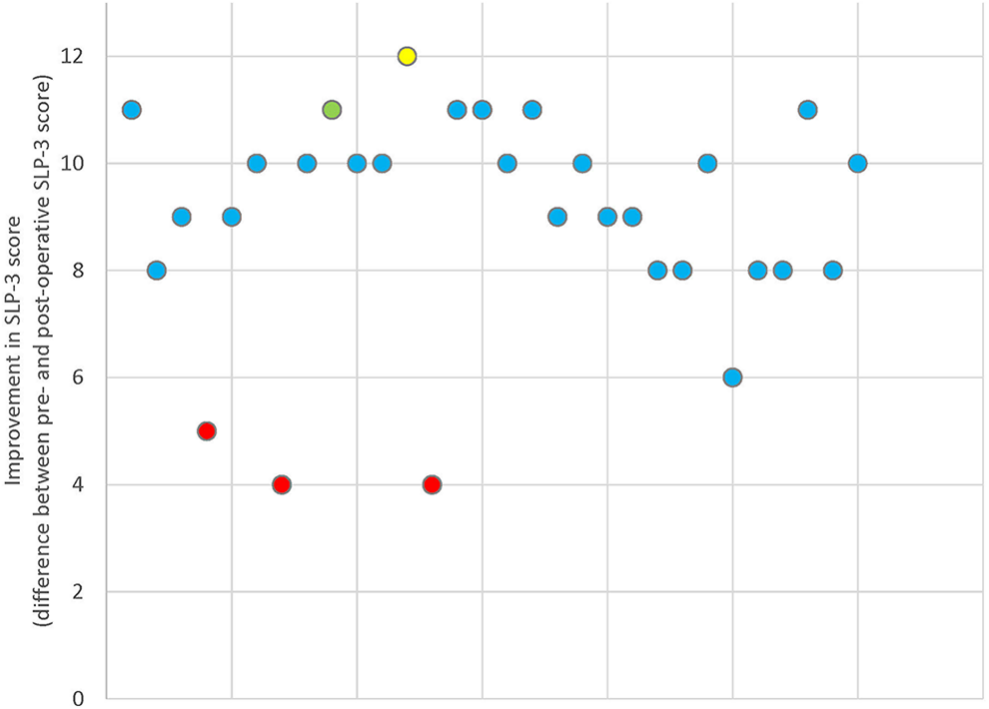
Scatter plot showing individual patients’ improvements in speech scores following surgery. Patients with inadequate postoperative speech therapy (red) and surgical complications are highlighted, including palatal fistulas (yellow) and minor flap dehiscence (green).

Individual patient improvements in speech score following surgery are summarized in Figure 3. Patients who did not receive sufficient speech therapy postoperatively (n = 3) improved significantly less than their counterparts (*P* < .001). Other independent variables tested for significant effect on speech score changes were age, syndromic versus nonsyndromic etiology, and a history of previous surgery. However, these variables did not show a significant effect (*P* = .38, *P* = .9, and *P* = .35 respectively).

Minor complications were found, with 7 (23%) patients reporting snoring symptoms, which resolved in 6 patients by 6 months postoperatively; none required flap takedown. One patient developed palatal partial dehiscence at flap inset site, which healed without need for surgical intervention. One patient presented postoperatively with a dehiscence of the posterior pharyngeal wall donor site, which was uncomplicated and required no intervention, healing spontaneously. There were no cases of infection, no cases of feeding difficulties that required nasogastric feeds or a prolonged hospital stay. Major complications included 1 (3%) patient with persistent snoring for whom polysomnography revealed mild OSA, not requiring flap takedown, and 1 (3%) patient developed a fistula that required reoperation for fistula closure. There were no cases of flap dehiscence requiring revision, airway obstruction requiring flap takedown, hemorrhage, intensive care unit (ICU) admission, reintubation, readmission, or mortality.

Median skin-to-skin operative time was 73.5 minutes (range 55-95 minutes), for patients undergoing pharyngeal flap procedure alone (n = 18). Median length of stay (LOS) was 50.3 hours (range 25.8-141.3).

## Discussion

### Outcomes/Complications

#### Speech

We achieved improvement in speech in all patients as measured on the SLP-3 speech scale. Velopharyngeal competence was fully restored in 83% patients, and borderline competence was found in 10% of patients postoperatively. Despite some improvement, 3 patients with cleft lip and palate had unsatisfactory functional speech results, which may be due to inadequate speech therapy postoperatively. Further, although these 3 patients did not arrive at complete resolution, these and all patients in our series did see improvement, as indicated in Figure 2 (improvement per subgroup). Our previous study offered similar outcomes, albeit with different inclusion criteria (Arneja et al., 2008). We specifically excluded patients with 22qDS from this series in an effort to determine whether other groups would be amenable to benefit from this technique.

Other groups have found similar results. A similar through-and-through dissection of the soft palate was used by Elsheikh and El-Anwar. (2016) who showed improvement in VPI in a series of 12 patients, mostly with postcleft VPI. Emara and Quriba (2012) also used through-and-through dissection of the soft palate for inset of their L-shaped flap to treat VPI in a series of 26 postpalatoplasty patients and showed restoration of normal velopharyngeal competence in 73% of patients, with 19% of patients having borderline sufficiency, and only 8% being borderline insufficient. This group also used direct visualization by endoscope to aid in flap inset.

#### Complication rates

Pharyngeal flaps are the most commonly reported procedure for VPI (49% of studies), with the highest rates of OSA (5.1%) and lowest rates of revisional surgery required (6.1%; de Blacam et al., 2018). A recent study using the 2012 American College of Surgeons National Surgical Quality Improvement Program Pediatric database identified the overall perioperative complication rate for posterior pharyngeal flap surgery to be low (5.3%) and identified patients with underlying cardiac risk factors, severe American Society of Anesthesiologists Physical Status class, and asthma as having heightened risk profiles (Swanson et al., 2016) Our major complication rate of 3% OSA and 3% rate of reoperation compare favorably to these studies in the literature. None of our patients required additional procedures for their VPI, although 1 patient did require a fistula repair.

Acute and possibly life-threatening postoperative complications such as ascending meningitis, life-threatening bleeding, sleep apnea, and airway compromise were not found in our series; Hogan (1973) suggested that mandatory postoperative management of the airway is necessary to prevent airway complications. Some even suggest mandatory post-op ICU admissions (Reddy et al., 2017). In our study, we had no acute postoperative complications, with no ICU admissions and no readmissions postdischarge.

In our study, rates of perioperative complication were mild OSA (3%), posterior wall dehiscence (3%), and fistula (3%), with no cases of flap dehiscence or acute respiratory problems. Some have suggested that pharyngeal flaps have previously required more revision (29%) than sphincter and Furlow procedures (Bohm et al., 2014); we did not note this in our study.

### Technical Details and Modifications

The technique currently employed at our center is largely similar to a previous publication on this subject (Arneja et al., 2008) with some subtle modifications. The posterior pharyngeal donor site is closed in order to minimize a demucosalized surface and also to help further obturate the central velopharynx. Closure of the donor site can decrease postoperative pain, permitting earlier return to oral intake, narrowing the posterior pharyngeal wall, and although not studied formally, potentially assisting in reduced airflow escape by this narrowing, and maintaining sphincteric action of the pharyngeal wall (Boutros & Cutting, 2013).

Additionally, nasal stents are not currently used to avoid postoperative synechiae between the raw surfaces of the lateral pharyngeal ports, since we primarily close the posterior pharyngeal wall. Stents could afford some degree of airway protection; however, in our current series, no patients had airway compromise in the acute setting. In our experience, there has been no clinical scenario that the use of this technique cannot be considered. The palatal back cut 5 mm posterior to the hard–soft junction is safe and easy for inset as long as there is ∼15 mm+ of soft palate posterior to the hard–soft junction. Furthermore, prior to embarking on this procedure, in an effort to ensure the airway is not compromised postoperatively, if patients had 2+ tonsillar hyperplasia or greater, a tonsillectomy was performed preoperatively and pharyngeal flap surgery performed 6 months posttonsillectomy. Compared to other techniques, the technique currently described gives more respect to the local palatal anatomy with minimal dissection and less compromise of residual palatal physiology (Elsheikh & El-Anwar, 2016).

Finally, as described by Arneja et al. (2008), one of the main advantages of this modified through-and-through technique for the dissection of the soft palate is direct visualization and high inset of pharyngeal flap inset at the level the normal velopharyngeal port closes, which we believe leads to better outcomes. Additionally, given the absence of lining flaps that need to be developed and inset, this technique reduces dissection required and ultimately a short operative skin-to-skin time.

### Subgroup Analysis

We attempted to determine whether there were subgroups of patients who did not do as well with this technique; however, given our small subgroups within the larger operated pool of patients with VPI, there was little conclusions we could draw.

#### Patients with poor outcomes

An SLP-3 score of 3 or greater shows that velopharyngeal competence was not restored. Three (10%) patients achieved a score of 3 to 6, which is considered a borderline valving mechanism, and 2 (7%) patients scored 7 on the SLP-3 scale, falling into the category of incompetent valving mechanism. Of the 5 patients, 2 had coronal and 3 had circular closure patterns. Gap sizes were small (1), medium (2), and large (2), and lateral wall motion was none (2), minimal (2), and moderate (1). These heterogeneous anatomical findings do not seem to indicate influence on speech outcomes. Three patients from our cohort had persistent compensatory articulation errors requiring intensive speech therapy; however, they did not receive sufficient speech therapy. There were no other etiological or demographic trends or findings we could find amongst these 5 patients.

#### Syndromic patients

Eleven of our patients were found to have syndromic comorbidities, 3 of which had VPI as a result of a neurobehavioral syndrome. One syndromic child was noted to have snoring postoperatively, and one experienced dehiscence of the posterior wall, which healed spontaneously and no surgical intervention was necessary. Altogether, the median SLP-3 score of syndromic patients improved from 11 of 13 to 1 of 13, and only 2 noted poor outcomes, both having a neurobehavioral syndrome.

#### Outcomes based on closure pattern

Traditionally, the pharyngeal flap is preferred in patients with a mobile lateral pharyngeal wall and limited velar movement (Argamaso et al., 1980; Emara & Quriba, 2012), whereas a sphincter pharyngoplasty may be recommended in patients with poor lateral wall movement (Ekin et al., 2017); this sentiment is echoed in a recent review of VPI management (Naran et al., 2017). Armour et al. (2005) reported better results in circular and sagittal closure patterns when compared to coronal patterns in pharyngeal flap pharyngoplasty. Ekin et al. (2017), however, demonstrated no statistical difference between coronal versus noncoronal velopharyngeal gaps when measuring objective speech outcomes following pharyngeal flap surgery. Contrary to traditional belief about preoperative velopharyngeal dynamics, it has been shown that with sufficiently high flap inset, the modified pharyngeal flap restored velopharyngeal competence in a series of patients with 22qDS all of whom had minimal lateral pharyngeal wall movement preoperatively (Arneja et al., 2008). Few studies have looked specifically at the impact of closure pattern on pharyngeal flap outcomes (Armour et al., 2005; Abdel-Aziz et al., 2011; Ekin et al., 2017). In this present series, we demonstrate effective restoration of velopharyngeal competence in most patients; however, we did select patients for this technique based on preoperative closure pattern or the degree of lateral wall motion.

### Resource Utilization

Median skin-to-skin operative time was 73.5 minutes (range 55-95 minutes) for patients (60%) with exclusively pharyngeal flap procedure and 92.5 minutes (range 76-164) for patients (40%) who received an additional procedure at the time of the pharyngeal flap surgery. Many studies suggest keeping patients in the intensive care setting, which would increase case costs; all of our patients were admitted to a regular inpatient bed. Furthermore, a median 73.5 minutes of skin-to-skin surgical time is anecdotally considerably faster than the current accepted standard, the Hogan technique. A median of 50.3 hours LOS (range 25.8-141.3 hours) was noted after surgery, which compares favorably to a recent retrospective study of pharyngeal flaps at a tertiary center that reported a mean LOS of 65.4 hours (Chao et al., 2018). They identified modifiable factors that significantly affected LOS: time until oral feeding and time until adequate oral feeding postoperatively, duration of narcotics postoperatively, and the use of intraoperative antiemetics. Duration of anesthesia had a positive linear correlation with LOS. A study on ambulatory cleft lip repair has been previously published (Arneja & Mitton, 2013) and future study should be directed on strategies to reduce the LOS for palatal and pharyngeal surgeries.

The goal to achieve a “good (quality)–fast (time)–cheap (cost)” outcome exists as a strategic mantra in production, known as the *iron triangle* (Atkinson, 1999). At its foundation exists, the inherent paradox of developing a highly safe and efficacious product, while maintaining cost-effectiveness and efficient resource utilization. As surgeons, our surgical product’s outcomes should be measured with a similar framework. The technique described herein yields a low reoperation rate, significant improvements in speech outcome, while operative resource utilization is minimal, requiring a median operative time of 73.5 minutes (range 55-95), and a bottom-up microcosted case cost of CAD $5217.00 (Table 2). We would argue that by “setting the stage” with this baseline economic data, we can create a comparator for future groups to contrast against. The need for economic accountability in health care treatment paradigms is important in the determination of what surgical procedures to offer our patients and their impact on the system.

**Table 2.** Case Cost (Bottom-Up Microcosting Methodology, Not Including Physician {Surgeon, Anesthetist} Compensation).

Surgical case costs	
Operative time (median)	73.5 minutes
Cost/minute OR time	$22
Total surgical case cost	CAD $1617
Inpatient costs	
Nights in hospital	2
Cost/night in hospital	$1800
Total hospital case cost	CAD $3600
Total case cost	CAD $5217

## Limitations

Perceptual speech analysis is the gold standard for evaluating VPI; however, there is currently no standardized evaluation tool or protocol that is routinely used or reported within the literature (Ruda et al., 2012). This creates a major problem with the treatment of VPI in that lack of a consistent tool to report/compare postoperative speech outcomes results in difficulty in comparing research in the literature objectively, therein rendering it difficult to draw general conclusions about success rates of pharyngeal flaps (Armour et al., 2005). We chose to replicate the same methodology as it is related to outcomes through using the same SLP-3 scoring system in our previous research (Arneja et al., 2008) and in this present research. Additionally, nasometry is routinely performed at our institution preoperatively, but not routinely performed postoperatively. It is not a component of the SLP-3 speech rating scale but should be considered for future research outcome studies as an objective measurement tool. Furthermore, tubing is certainly a possibility postoperatively, given the absence of a lining flap; however, none of our patients had severe enough VPI postoperatively to require an additional nasoendoscopy wherein the flap could concurrently be visualized and examined. Other limitations in our study include the lack of a control group as well as a relatively small sample size, particularly representing the different etiologies of VPI.

## Conclusions

To build on the conclusions from previous papers, the technique described herein can be characterized as “good-fast-cheap” and offers specific advantages, including (1) direct visualization and placement of the flap at the desired cephalad location; (2) low risk of flap dehiscence and migration as the flap is sandwiched between the oral mucosa, levator muscle, and nasal mucosa; (3) objectively measured improvement of VPI using SLP-3; (4) suitable for all cases of VPI despite etiology; (5) low complication rate and no cases requiring intensive care admission; (6) velopharyngeal anatomy consistent with a moderate to large gap size, circular or coronal closure pattern; and (7) efficient resource utilization with a case cost of CAD $5217.00.
